# Supplementary material for: Quantitative proteomics defines mechanisms of antiviral defence and cell death during modified vaccinia Ankara infection
Source: Nat Commun. 2023 Dec 8;14:8134. doi: 10.1038/s41467-023-43299-8 (PMC10709566; doi:10.1038/s41467-023-43299-8)
Supplement: Supplementary file 2 — Description of Additional Supplementary Information [file 41467_2023_43299_MOESM2_ESM.pdf]

Title: Supplementary Data 1
Description: Interactive spreadsheet of all data in this manuscript. The “Plotter” worksheet enables generation of graphs for all the human and viral proteins quantified in HFFF-TERTs and/or THP-1 macrophages, and easy visualization of statistics. It also includes the temporal profiles of proteins quantified in HFFF-TERTs infected with vaccinia virus Western Reserve (VACV-WR) (published in Soday et al. 2019). The “Data” worksheet shows minimally annotated protein data, with only formatting and normalization modifying the raw data, and all derived p values. One-way ANOVA was used to estimate p-values comparing all indicated time points to mock samples. P-values were corrected for multiple hypothesis testing using the method of Benjamini-Hochberg.

Title: Supplementary Data 2
Description: Each worksheet lists the proteins identified after application of a series of filters that identified differential modulation by MVA infection of HFFF-TERTs and THP-1 macrophages. On the right, the results of functional enrichment analysis with the Database for Annotation, Visualization and Integrated Discovery (DAVID) software and the proteins in each enriched cluster are shown. P-values were estimated in DAVID software using Fisher’s Exact tests and were corrected for multiple hypothesis testing using the method of Benjamini-Hochberg.

Title: Supplementary Data 3
Description: Each worksheet lists the proteins identified after application of a series of filters that identified differential regulation of host protein abundance by MVA versus VACV-WR infection of HFFF-TERTs (worksheets A-F), or of MVA infection of HFFF-TERTs versus THP-1 macrophages (worksheets G-I). On the right, the results of functional enrichment analysis with the Database for Annotation, Visualization and Integrated Discovery (DAVID) software and the proteins in each enriched cluster are shown. P-values were estimated in DAVID software using Fisher’s Exact tests and were corrected for multiple hypothesis testing using the method of Benjamini-Hochberg.

Title: Supplementary Data 4
Description: MVA protein and transcriptional classes. A comparison to our prior quantitative proteomic analysis of VACV-WR infection (Soday et al. 2019) is also included in this table. Each generated four temporal classes of viral proteins and, as some of these data are discordant, a further column is included in the table indicating concordant temporal classes. Transcriptional classes and functional category information were derived from Yang et al., 2010 and Yang et al., 2015.

Title: Supplementary Data 5
Description: Raw data for all peptides quantified in this study.

Title: Supplementary Data 6
Description: Reporter ion isotopic distributions for TMT reagents used for the experiments in HFFF-TERTs (A) and THP-1 macrophages (B).

Title: Supplementary Data 7
Description: Complete comparison of the proteomic changes during MVA infection in HFFF-TERTs and THP-1 macrophages. To facilitate optimal comparison of data from different cell types, upregulation was defined as > 1.5 fold change and downregulation as < 0.5 fold change compared to mock for HFFF-TERTs, or > 2 fold change and < 0.5 fold change, respectively, for THP-1s.
