# Supplementary material for: Quantitative proteomics defines mechanisms of antiviral defence and cell death during modified vaccinia Ankara infection
Source: Nat Commun. 2023 Dec 8;14:8134. doi: 10.1038/s41467-023-43299-8 (PMC10709566; doi:10.1038/s41467-023-43299-8)
Supplement: Supplementary file 10 — Reporting Summary [file 41467_2023_43299_MOESM10_ESM.pdf]

nature portfolio

Corresponding author(s): *Double-anonymous peer review submissions: write DAPR and your manuscript number here instead of author names.*

Last updated by author(s): *YYYY-MM-DD*

Reporting Summary

Nature Portfolio wishes to improve the reproducibility of the work that we publish. This form provides structure for consistency and transparency in reporting. For further information on Nature Portfolio policies, see our Editorial Policies and the Editorial Policy Checklist.

Statistics

For all statistical analyses, confirm that the following items are present in the figure legend, table legend, main text, or Methods section.

n/a	Confirmed	
☐	☒	The exact sample size (*n*) for each experimental group/condition, given as a discrete number and unit of measurement
☐	☒	A statement on whether measurements were taken from distinct samples or whether the same sample was measured repeatedly
☐	☒	The statistical test(s) used AND whether they are one- or two-sided<br>*Only common tests should be described solely by name; describe more complex techniques in the Methods section.*
☒	☐	A description of all covariates tested
☐	☒	A description of any assumptions or corrections, such as tests of normality and adjustment for multiple comparisons
☐	☒	A full description of the statistical parameters including central tendency (e.g. means) or other basic estimates (e.g. regression coefficient) AND variation (e.g. standard deviation) or associated estimates of uncertainty (e.g. confidence intervals)
☐	☒	For null hypothesis testing, the test statistic (e.g. *F*, *t*, *r*) with confidence intervals, effect sizes, degrees of freedom and *P* value noted<br>*Give P values as exact values whenever suitable.*
☒	☐	For Bayesian analysis, information on the choice of priors and Markov chain Monte Carlo settings
☒	☐	For hierarchical and complex designs, identification of the appropriate level for tests and full reporting of outcomes
☐	☒	Estimates of effect sizes (e.g. Cohen's *d*, Pearson's *r*), indicating how they were calculated

Our web collection on statistics for biologists contains articles on many of the points above.

Software and code

Policy information about availability of computer code

Data collection	The following softwares were used for data collection:<br>1. Attune NxT Software (version 3.1.2);<br>2. IncuCyte 2021A
Data analysis	The following softwares were used for data analysis:<br>1. FlowJo (version 10.6.1);<br>2. A Sequest-based in-house software pipeline for quantitative proteomics (constituent components published as described in 'Code Availability');<br>3. XLStat premium 2021.2.2 (Addinsoft);<br>4. DAVID software (https://david.ncifcrf.gov/, version 6.8);<br>5. Cluster 3.0;<br>6. Java Treeview 1.2.0 (http://jtreeview.sourceforge.net);<br>7. Perseus (version 1.5.1.6);<br>8. Incucyte Base Analysis Software v2021A;<br>9. Imaris 10.0 (Oxford Instruments);<br>10. GraphPad Prism (version 10.0.2)

For manuscripts utilizing custom algorithms or software that are central to the research but not yet described in published literature, software must be made available to editors and reviewers. We strongly encourage code deposition in a community repository (e.g. GitHub). See the Nature Portfolio guidelines for submitting code & software for further information.

Data

Policy information about availability of data

All manuscripts must include a data availability statement. This statement should provide the following information, where applicable:

- Accession codes, unique identifiers, or web links for publicly available datasets
- A description of any restrictions on data availability
- For clinical datasets or third party data, please ensure that the statement adheres to our policy

The proteomic data generated in this study have been deposited in the ProteomeXchange Consortium via the PRIDE partner repository under accession code PXD039034. Furthermore, all peptides quantified in this study are provided in Supplementary Table 5. Source data are provided with this paper, and source data for all line graphs can be found in Supplementary Table 1. All materials described in this manuscript, and any further details of protocols employed can be obtained on request from the corresponding authors by email to jonas.albarnaz@pirbright.ac.uk or mpw1001@cam.ac.uk. Source data are provided with this paper.
A combined database was constructed from the human database (accessed 20th June 2022, UP000005640), and the MVA proteome (accessed 20th June 2022, UP000172909), both accessed on Uniprot database.
The human sequences for GLE1 (NM_001003722.2:88-2184), ISG20 (NM_002201.6:86-631), NSA2 (NM_014886.6:113-895]), NUP54 (NM_017426.4:24-1547), NUP62 (NM_153719.4:412-1980), NUP88 (NM_001320653.2:13-2286), OASL (NM_003733.4:276-1820), RBM28 (NM_018077.3:116-2395), and ZNFX1 (NM_021035.3:86-5842) are available under the given accession code on NCBI.

Research involving human participants, their data, or biological material

Policy information about studies with human participants or human data. See also policy information about sex, gender (identity/presentation), and sexual orientation and race, ethnicity and racism.

Reporting on sex and gender	N/A
Reporting on race, ethnicity, or other socially relevant groupings	N/A
Population characteristics	N/A
Recruitment	N/A
Ethics oversight	N/A

Note that full information on the approval of the study protocol must also be provided in the manuscript.

Field-specific reporting

Please select the one below that is the best fit for your research. If you are not sure, read the appropriate sections before making your selection.

☒ Life sciences ☐ Behavioural & social sciences ☐ Ecological, evolutionary & environmental sciences

For a reference copy of the document with all sections, see nature.com/documents/nr-reporting-summary-flat.pdf

Life sciences study design

All studies must disclose on these points even when the disclosure is negative.

Sample size	For the proteomics experiments, sample size was determined based on the original study describing quantitative temporal viromics (PMID: 24906157) as well as previous studies of similar nature.<br>Gain-of-function screen experiments were carried out with n = 2 biological replicates (2 separate wells per condition in 24-well plates).<br>Lactate dehydrogenase (LDH) release assays were carried out with n = 3 biological replicates (3 separate wells per condition in 96-well plates).<br>Fluorescence microscopy experiments were carried out with n = 2 biological replicates (2 separate wells per condition in 12-well plates).<br>Immunoblot quantification was from 2 independent experiments. Sample size was chosen to provide statistical information about data reproducibility.
Data exclusions	For the gain-of-funtion screen, images acquired on Incucyte S3 containing bright fluorescent artifacts from the first scan or rare MVA-GFP plaques were excluded from analysis because they skewed the calculation of GFP area.<br><br>In the remaining analyses, no data exclusions were made.
Replication	The quantitative temporal viromics of the time course infection with MVA was carried out twice with one sample analysed per time and conditions as described in the methods section. Quantitative temporal viromics of VACV-WR was published elsewhere (PMID: 31067474) and re-analysed for comparison with MVA.<br>Gain-of-function screen experiments were carried out 4 times.<br>Lactate dehydrogenase (LDH) release assays with THP-1 cells were carried out 3 times.<br>Inflammasome fluorescence microscopy and GSDMD immunoblotting experiments were carried out twice each.

All attempts at replication were successful.

Data shown in Extended Data Figures 1, 4, and 8a are from experiments carried out once.

Randomization

Each experiment was carried out separately, without randomization.
Randomization is not applicable because experimental and control samples are allocated side by side to minimize unpredictable environmental variations.

Blinding

No blinding was applied as all the assays were in vitro based and we did not include human subjects/samples in this study.

Reporting for specific materials, systems and methods

We require information from authors about some types of materials, experimental systems and methods used in many studies. Here, indicate whether each material, system or method listed is relevant to your study. If you are not sure if a list item applies to your research, read the appropriate section before selecting a response.

Materials & experimental systems

n/a	Involved in the study	
☐	☒	Antibodies
☐	☒	Eukaryotic cell lines
☒	☐	Palaeontology and archaeology
☒	☐	Animals and other organisms
☒	☐	Clinical data
☒	☐	Dual use research of concern
☒	☐	Plants

Methods

n/a	Involved in the study	
☒	☐	ChIP-seq
☐	☒	Flow cytometry
☒	☐	MRI-based neuroimaging

Antibodies

Antibodies used

1. Rabbit polyclonal anti-VACV (Laboratory of Geoffrey L. Smith, PMID: 11162827);
2. Rabbit polyclonal anti-FLAG (Cell Signaling Technology, #2368);
3. Rabbit polyclonal anti-CANX (LSBio, LS-B6881);
4. Mouse monoclonal anti-GAPDH 6C5 (Millipore, MAB374);
5. Rabbit monoclonal anti-GSDMD E504N (Cell Signaling Technology, #69469);
6. IRDye 800CW-conjugated goat anti-rabbit IgG (LICOR, 926-32211);
7. IRDye 680LT-conjugated goat anti-mouse IgG (LICOR, 926-68020);
8. Rabbit polyclonal anti-ASC (AdipogGen, AL177);
9. Alexa Fluor 568-conjugated goat anti-rabbit IgG (Invitrogen, A11011);
10. Alexa Fluor 488-conjugated goat anti-rabbit IgG (Abcam, ab150077).

Validation

The antibody (1) was validated in PMID: 11162827, whilst the remaining antibodies were validated by their respective manufacturers and are available commercially. Please see below the links:
Antibody (2): https://www.cellsignal.com/products/primary-antibodies/dykddddk-tag-antibody-binds-to-same-epitope-as-sigma-aldrich-anti-flag-m2-antibody/2368
Antibody (3): https://www.lsbio.com/targets/canx-calnexin/g799
Antibody (4): https://www.sigmaaldrich.com/GB/en/product/mm/mab374af488?gclid=CjwKCAjwp8OpBhAFEiwAG7NaEvAGuZzZGBWMFuT8dd-fwZ-fvSdFbEdrte_X53Q-O3H1oylYu2bqiRoCdooQAvD_BwE
Antibody (5): https://www.cellsignal.com/products/primary-antibodies/gasdermin-d-e5o4n-rabbit-mab/69469
Antibody (6): https://www.licor.com/bio/reagents/irdye-800cw-goat-anti-rabbit-igg-secondary-antibody
Antibody (7): https://www.licor.com/bio/reagents/irdye-680lt-goat-anti-mouse-igg-secondary-antibody
Antibody (8): https://adipogen.com/ag-25b-0006-anti-asc-pab-al177.html/
Antibody (9): https://www.thermofisher.com/antibody/product/Goat-anti-Rabbit-IgG-H-L-Cross-Adsorbed-Secondary-Antibody-Polyclonal/A-11011
Antibody (10): https://www.abcam.com/products/secondary-antibodies/goat-rabbit-igg-hl-alexa-fluor-488-ab150077.html#:~:text=Triton%20X%2D100.-,ab150077%2C%20an%20Alexa%20Fluor%C2%AE%20488%2Dconjugated%20goat%20anti%2D,%2F1000)%20were%20also%20used.

Eukaryotic cell lines

Policy information about cell lines and Sex and Gender in Research

Cell line source(s)

HFFF-TERTs were described in PMID: 11257191, and were originally isolated from a white male 14-week old embryo, THP-1 monocytes were from ATCC (TIB-202) and the chicken embryo fibroblasts (CEFs) were obtained from the Pirbright Institute (Woking, UK). HEK 293T were purchase from ATCC (CRL-11268).

Authentication

HFFF-TERTs have been tested at regular intervals since isolation to confirm both that the HLA and MICA genotypes, and the

	morphology and antibiotic resistances are consistent with the original cells described in PMID: 11257191. In addition, HFFF-TERTs are routinely infected with the human cytomegalovirus Merlin strain, which is only permissive in human fibroblasts (dermal or foreskin), further limiting the chances that the cells have been contaminated with another cell type. CEFs were used to prepare working stocks of MVA, which grows to high infectious titres only in chicken embryo fibroblasts. THP-1 monocytes grew in suspension and differentiated into adherent macrophages upon treatment with 20 ng/ml PMA (phorbol 12-myristate 13-acetate) for 48 h. The cell line was purchased from ATCC, and validated by them. A THP-1 derivative cell expressing caspase-1 CARD fused to EGFP (C1C-EGFP) was validated previously by Florian Schmitt in PMID: 36315050. HFFF-TERTs overexpressing GLE1 (NM_001003722.2:88-2184), ISG20 (NM_002201.6:86-631), NSA2 (NM_014886.6:113-895), NUP54 (NM_017426.4:24-1547), NUP62 (NM_153719.4:412-1980), NUP88 (NM_001320653.2:13-2286), OASL (NM_003733.4:276-1820), RBM28 (NM_018077).3:116-2395), and ZNFX1 (NM_021035.3:86-5842) were validated with an anti-FLAG antibody using Flow Cytometry and Western Blot. HEK 293T (ATCC CRL-11268) were validated by ATCC before purchase.
Mycoplasma contamination	All cells used have regularly tested negative for mycoplasma.
Commonly misidentified lines (See ICLAC register)	No commonly misidentified cell line was used.

Plants

Seed stocks	N/A
Novel plant genotypes	N/A
Authentication	N/A

Flow Cytometry

Plots

Confirm that:

☒ The axis labels state the marker and fluorochrome used (e.g. CD4-FITC).

☒ The axis scales are clearly visible. Include numbers along axes only for bottom left plot of group (a 'group' is an analysis of identical markers).

☒ All plots are contour plots with outliers or pseudocolor plots.

☒ A numerical value for number of cells or percentage (with statistics) is provided.

Methodology

Sample preparation	MVA-infected HFFF-TERTs or PMA-differentiated THP-1 macrophages were detached with trypsin-EDTA (Gibco) 24 h post-infection, passed through a 70-µm cell strainer, washed in PBS and stained with Zombie Violet viability dye (BioLegend) for 30 min at 4°C in the dark. Cells were collected by centrifugation, washed once with PBS and fixed in Cytofix/Cytoperm fixation and permeabilization solution (BD Biosciences) for 30 min at 4°C in the dark. Cells were washed twice with Perm/Wash buffer (BD Biosciences) and stained with a polyclonal rabbit anti-VACV antibody68 diluted 1:500 in Perm/Wash buffer, followed by AlexaFluor 568-conjugated goat anti-rabbit IgG (Invitrogen) diluted 1:100. Alternatively, transduced HFFF-TERTs were harvested as described above and stained with rabbit anti-FLAG (Cell Signaling Technology, #2368) diluted 1:200 and AlexaFluor 488-conjugated goat anti-rabbit IgG (Abcam) diluted 1:100. Stained cells were suspended in PBS before data acquisition with an Attune NxT flow cytometer. Data were analysed with FlowJo software.
Instrument	Attune NxT flow cytometer, operated with Attune NxT Software (version 3.1.2).
Software	FlowJo (version 10.6.1).
Cell population abundance	Flow-assisted cell sorting was not used.
Gating strategy	A minimum 10,000 events was recorded and FSC-A by SSC-A was used to initially gate HFFF-TERTs or THP-1 macrophages. FSC-A by FSC-H was used in this gate to determine the single-cell population. FSC-A by Zombie Violet viability was used in this gate to determine the viable population. FSC-A by the desired immunostaining was used in the single-cell, viable population to determine the percentage of positive cells. As controls, the same gating strategy was applied to the single-cell, viable population of non-infected cells or parental cells, which were used to define the boundaries between "VACV-positive" and "VACV-negative" cells or "FLAG-positive" and "FLAG-negative" cells, respectively.

☒ Tick this box to confirm that a figure exemplifying the gating strategy is provided in the Supplementary Information.
