# Supplementary material for: Quantitative proteomics defines mechanisms of antiviral defence and cell death during modified vaccinia Ankara infection
Source: Nat Commun. 2023 Dec 8;14:8134. doi: 10.1038/s41467-023-43299-8 (PMC10709566; doi:10.1038/s41467-023-43299-8)
Supplement: Supplementary file 11 — Supplementary Figure [file 41467_2023_43299_MOESM11_ESM.pdf]

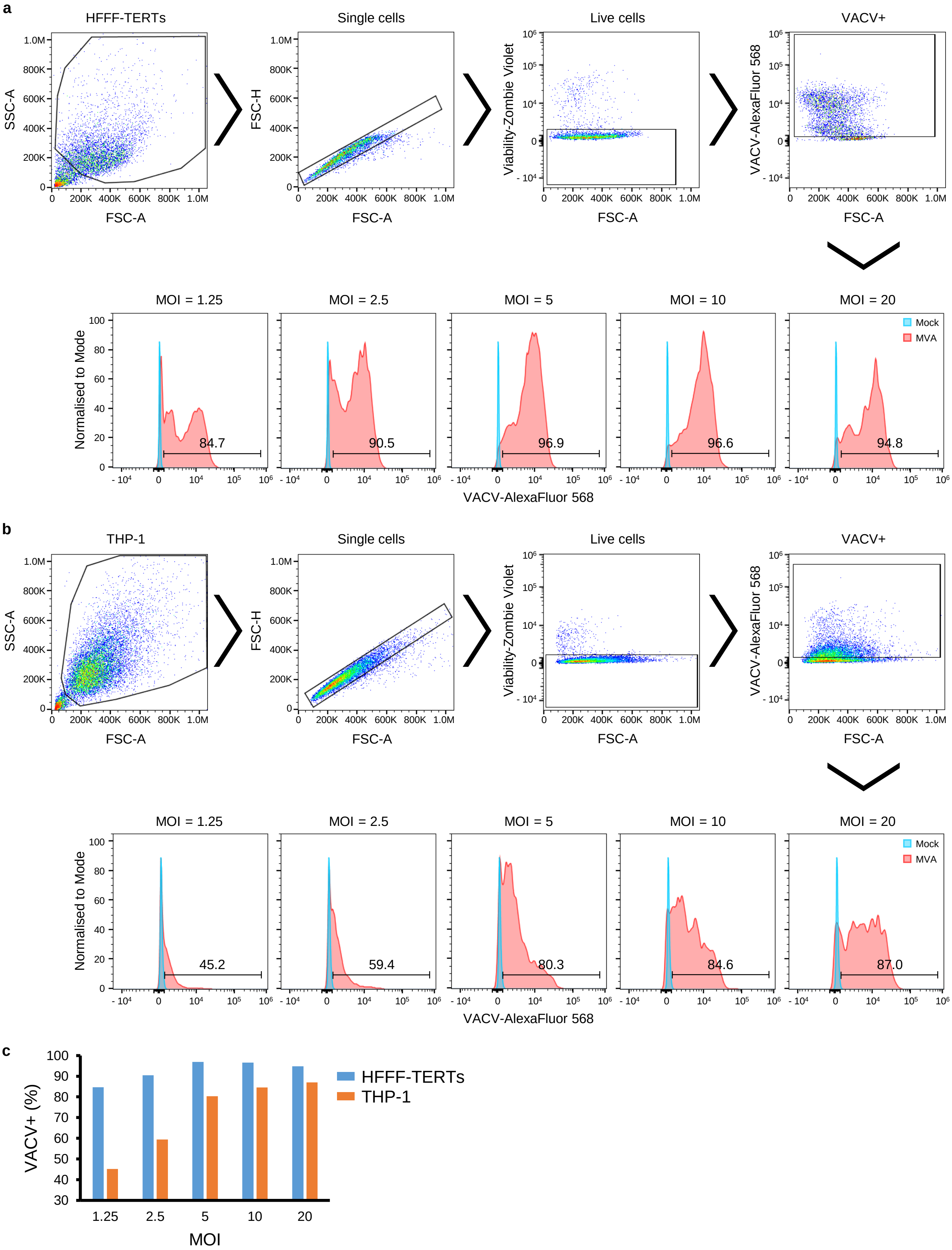

Supplementary Figure 1 | Percentage of cells expressing viral proteins at different MOIs. HFFF-TERTs (**a**) or PMA-differentiated THP-1 cells (**b**) were infected at the indicated MOI for 18 h, then stained with a polyclonal rabbit anti-VACV antibody. Top panels depict the gating strategy with representative plots of cells infected at the MOI of 1.25. **c** Percent of infection from HFFF-TERTs and THP-1 cells infected at different MOIs.

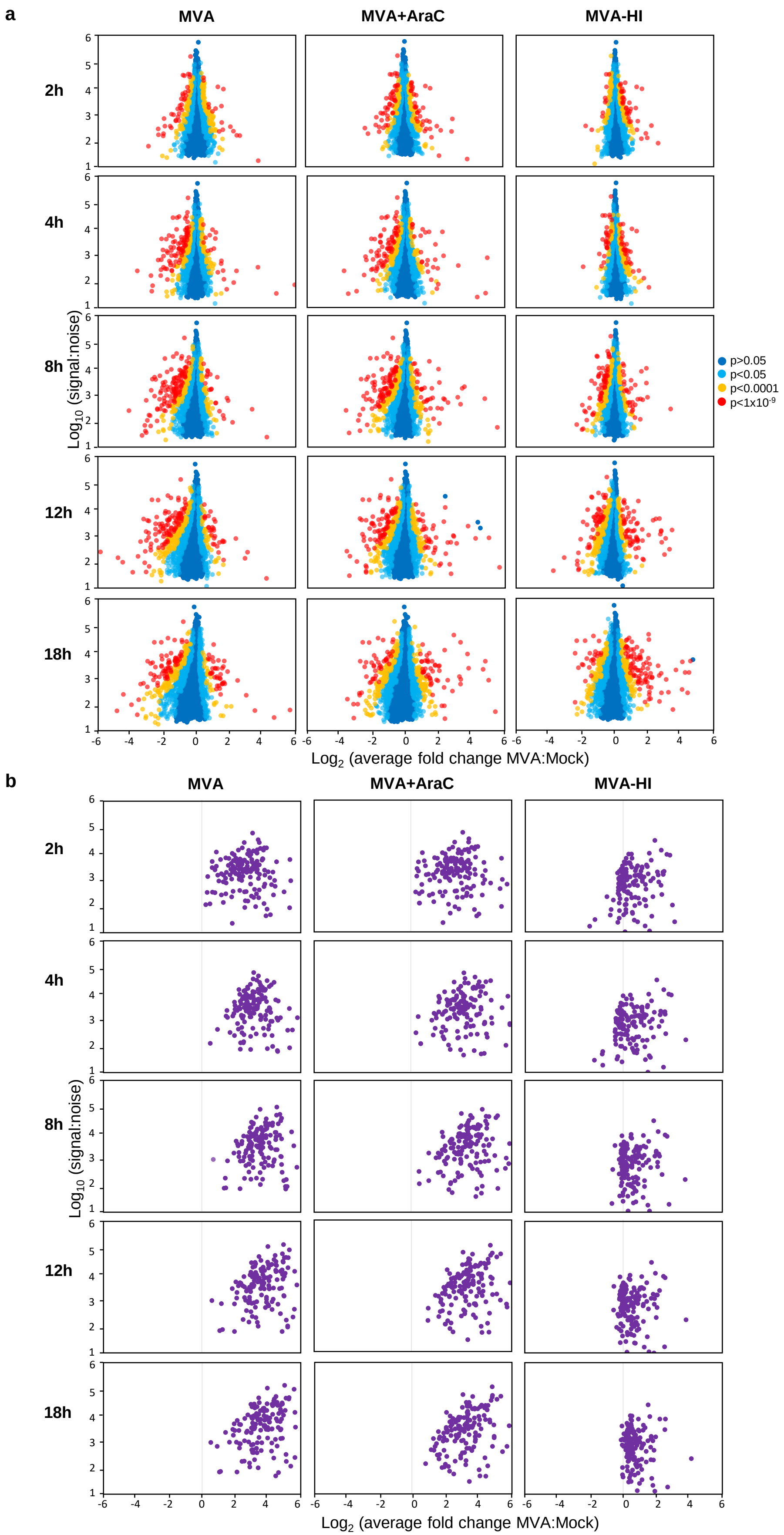

Supplementary Figure 2 | Dot plots of all proteins quantified. Average fold change was calculated from each of two biological replicates. **a** Human proteins. Significance B was used to estimate p-values that were adjusted for multiple hypothesis testing using the method of Benjamini-Hochberg[56,57]. The y-axis represents log_{10}(summed MS3 TMT intensities for all peptides from a given protein / average noise). **b** Viral proteins; p-values not displayed. Here, for the purposes of comparison between different types of sample or between different time points, the mock sample was used to calculate fold change.

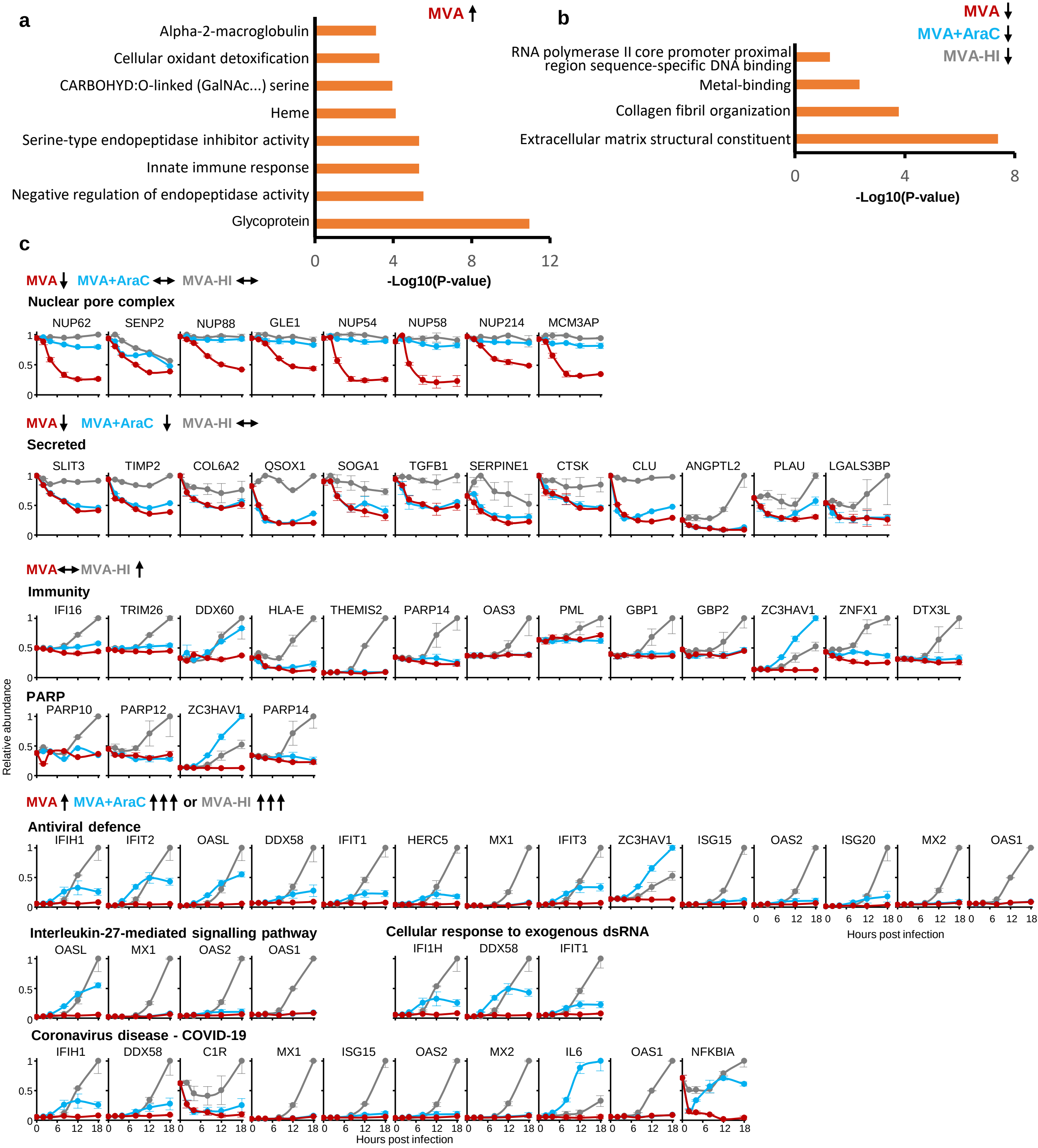

d

	MVA	↓	↓	↔	↑
	MVA+AraC	↔	↓		↑↑↑
	MVA-HI	↔	↔	↑	↑↑↑
Graph	Observed	0.0195	0.0011	-0.0003	0.0022
Assortativity	Random*	-0.0001 ± 0.0022	-0.0001 ± 0.0023	-0.0002 ± 0.0023	-0.0002 ± 0.0023
	Z-Score	8.75	0.563	-0.05	1.005
	P-Value	<1e-20	0.575	0.958	0.315

* Mean +/- SD for 1000 random samples

Supplementary Figure 3 | a-b Functional enrichment analysis of proteins regulated as indicated by MVA. **c** Graphical representation of protein expression over time for all pathways from **Figure 2c**. Error bars = range. **d** Graph assortativity of constituent proteins from each pathway, which quantifies the tendency of proteins in a group to interact preferentially with each other. Monte Carlo simulations employing 1000 randomized protein sets of equal size revealed that proteins decreased upon MVA infection and unchanged in the MVA + AraC and MVA-HI samples showed a highly significant tendency to interact.

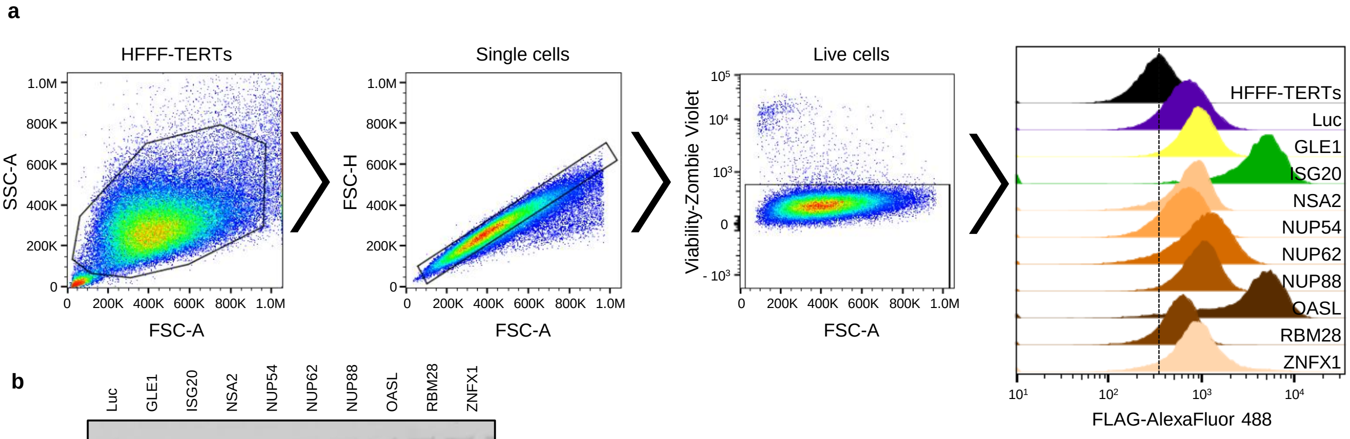

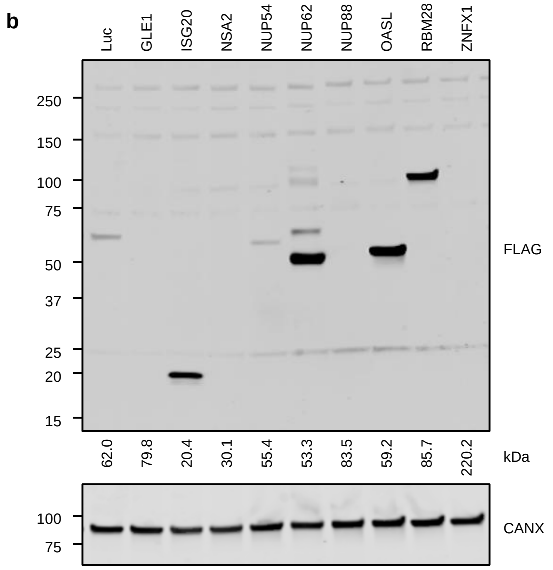

Supplementary Figure 4 | Validation of HFFF-TERT cell lines overexpressing selected human proteins. a Flow cytometry analysis of cells after intracellular staining with anti-FLAG antibody and AlexaFluor 488-conjugated secondary antibody. Panels on the left indicate the gating strategy. FLAG expression for each cell line is shown on the rightmost panel in comparison to parental HFFF-TERT cells. Luc = Luciferase control. **b** Immunoblotting analysis of the expression of FLAG-tagged proteins and calnexin (CANX), used as loading control. The positions of molecular mass markers in kDa are shown on the left of immunoblots and predicted molecular masses for each protein are shown below the upper immunoblot. Expression of GLE1, NSA2, and ZNFX1 could only be detected by intracellular flow cytometry.

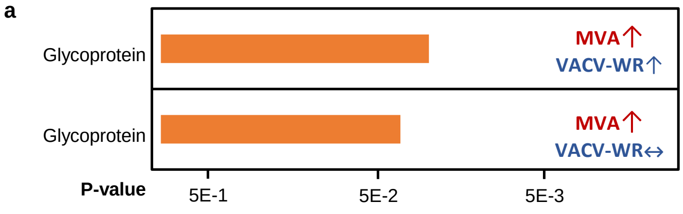

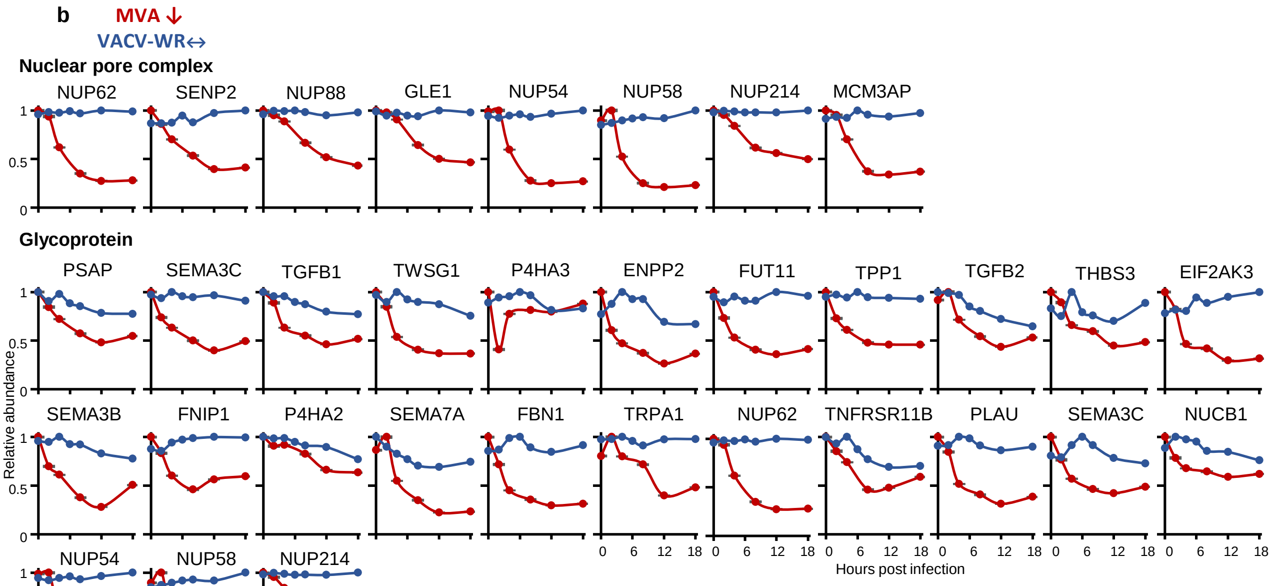
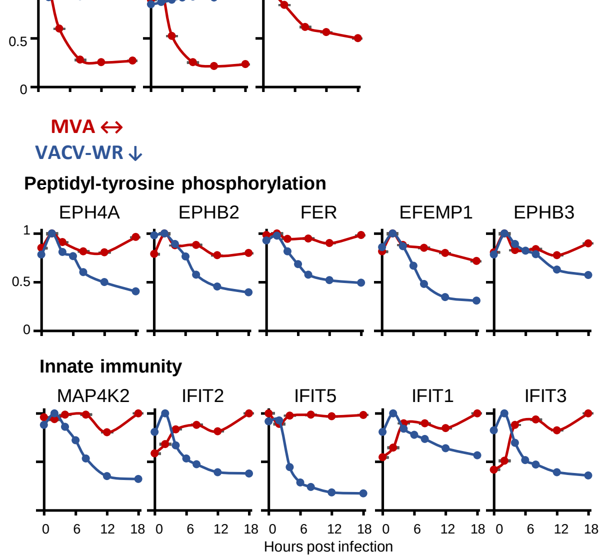

c

MVA	VACV	Graph Assortativity Observed	Random*	Z-Score	P-Value
↓	↔	0.017	-0.0002 ± .0024	7.19	6.31E-13
↑	↔	0.004	-0.0003 ± 0.0023	2.08	0.0374
↔	↓	0.0124	-0.0002 ± 0.0023	5.64	1.66E-08
↔	↑	-0.0013	0.0001 ± 0.0022	-0.51	0.604
↓	↓	-0.0002	-0.0003 ± 0.0024	0.022	0.983
↑	↑	-0.0003	-0.0001 ± 0.0020	-0.114	0.909

* Mean ± SD for 1000 random samples

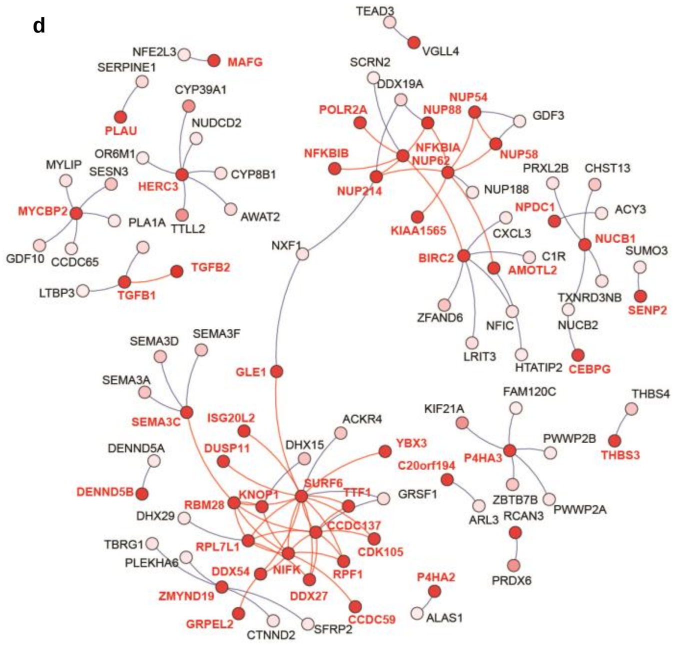

Supplementary Figure 5 | a Functional enrichment analysis of proteins regulated as indicated by MVA and VACV-WR. Only the term 'Glycoprotein' was enriched in each case; the majority of significantly enriched terms were found for proteins downregulated during MVA infection. **b** Graphical representation of protein expression over time for all pathways from **Figure 3c**. Error bars = range. **c** Graph assortivity of proteins regulated by MVA and VACV as indicated. **d** Interactions of proteins that decrease upon MVA infection but remain unchanged upon VACV-WR infection (red). For context, a subset of neighbouring proteins are displayed based on their proximity as assessed via random walk with restart (see Methods). For clarity, only proteins that interact with at least one additional protein in this subnetwork are displayed. Proteins degraded following MVA infection, but not VACV-WR, infection included nucleoporins and associated proteins, as well as TGFB1/2, NFKBIA/B, and a cluster of interacting nucleolar proteins.

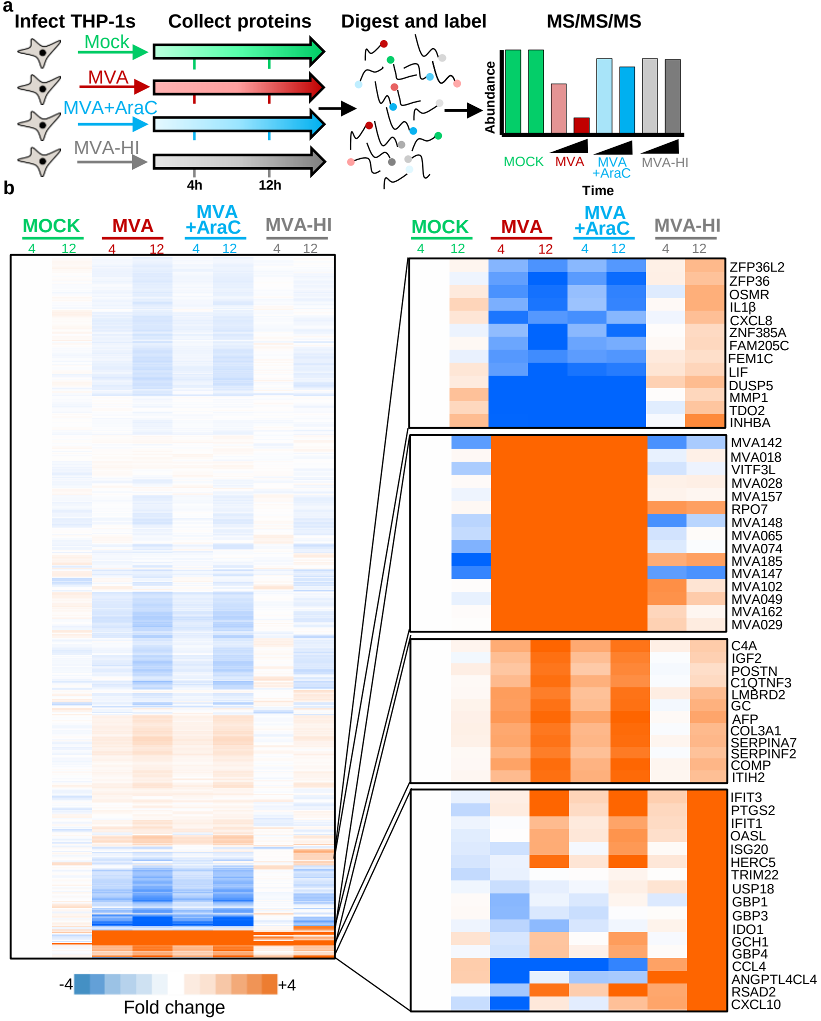

Supplementary Figure 6 | Quantitative temporal proteomic analysis of MVA infection in THP-1 human macrophages. a Schematic indicating the experimental workflow. **b** Hierarchical clustering of all proteins quantified in the two biological repeats. An enlargement is shown indicating groups of proteins that were significantly down- or upregulated during the course of the experiment.

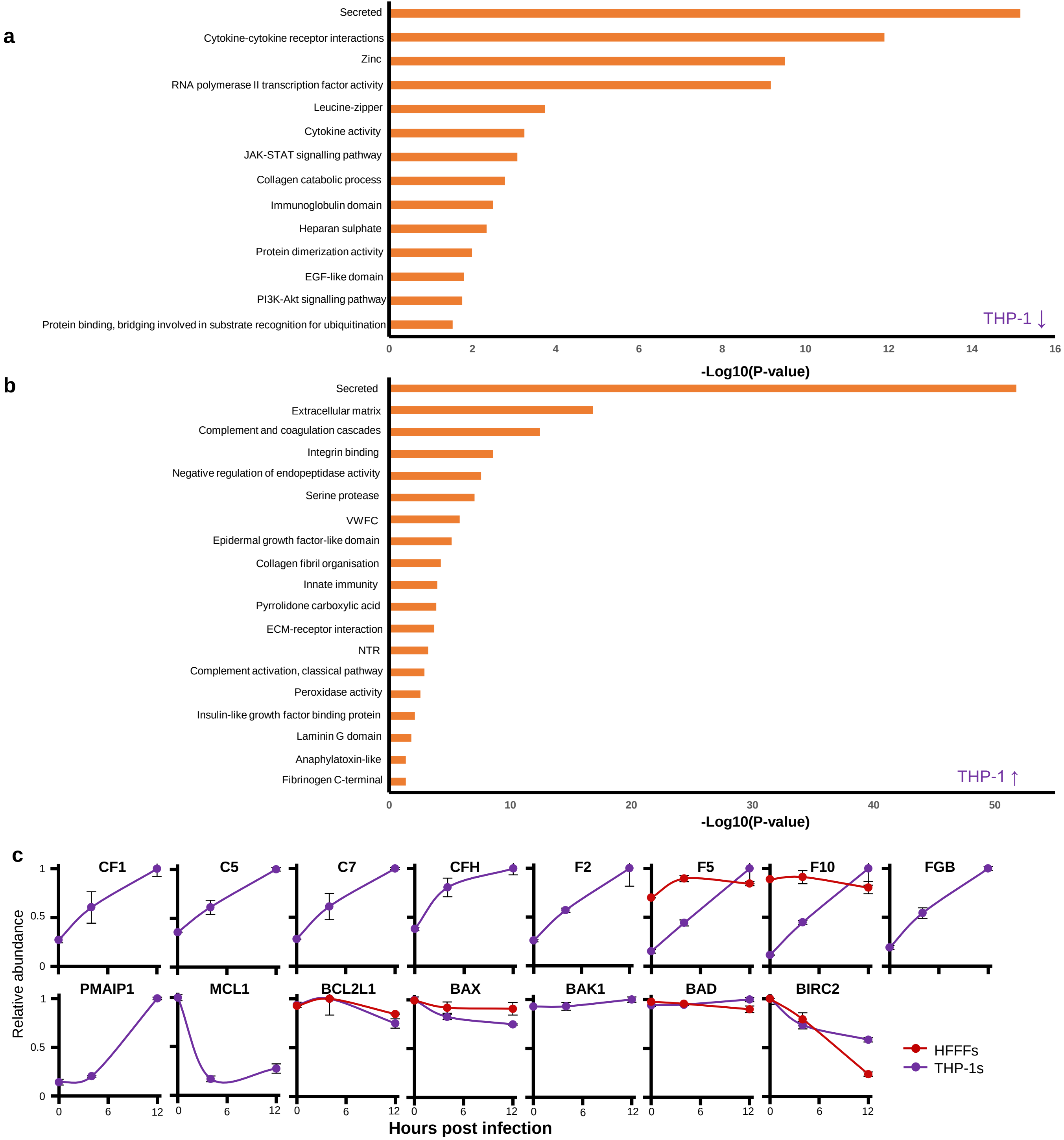

Supplementary Figure 7 | a Functional enrichment analysis of proteins downregulated >2 fold at any time point by MVA in THP-1 cells. **b** Functional enrichment analysis of proteins upregulated >2-fold at any time point by MVA in THP-1 cells. **c** Continued from **Figure 4a**. Examples of proteins regulated during MVA infection.

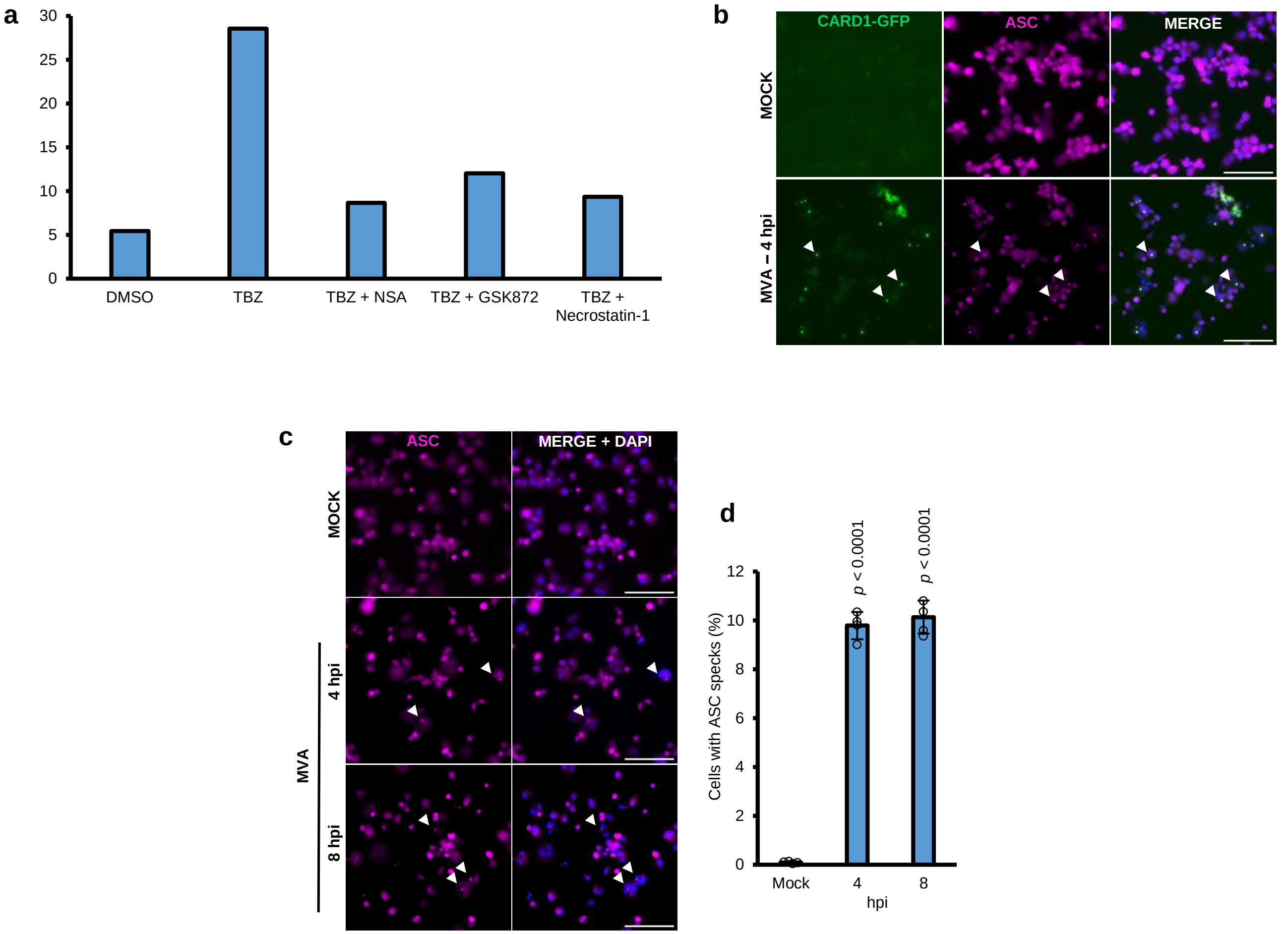

Supplementary Figure 8 | MVA infection induces pyroptosis in THP-1 cells. a To verify that the necroptosis inhibitors (NSA, GSK872, necrostatin-1) were active at the concentrations used for **Figure 4b**, HFFF-TERTs were treated with the indicated inhibitors in presence of the necroptosis stimulus of 30 ng/ml TNF-α, 5 µM BV-6 and 25 µM zVAD-fmk (TBZ) for 24 h, followed by lactate dehydrogenase (LDH) release assay. Means (*n* = 3 biological replicates) are shown. **b** PMA-differentiated THP-1 cells that ectopically express CARD1-GFP (THP-1$^{C1C\text{-}GFP}$) were infected with MVA (MOI = 5) for 4 h in presence of caspase-1 inhibitor VX-765 (8-h infection shown in **Figure 4c**). **c** Differentiated THP-1 were infected with MVA (MOI = 5) for 4 and 8 h in presence of caspase-1 inhibitor VX-765. Representative micrographs show caspase-1 CARD-GFP (green), ASC immunostaining (magenta) and DAPI staining (blue). Scale bar = 100 µm. Arrowheads indicate caspase-1/ASC specks. **d** Quantification of cells with ASC specks from **c**. Means + s.d. (*n* = 4 biological replicates from 2 independent experiments) are shown. Significance calculated using one-way ANOVA followed by post-hoc Dunnett's multiple comparisons test.

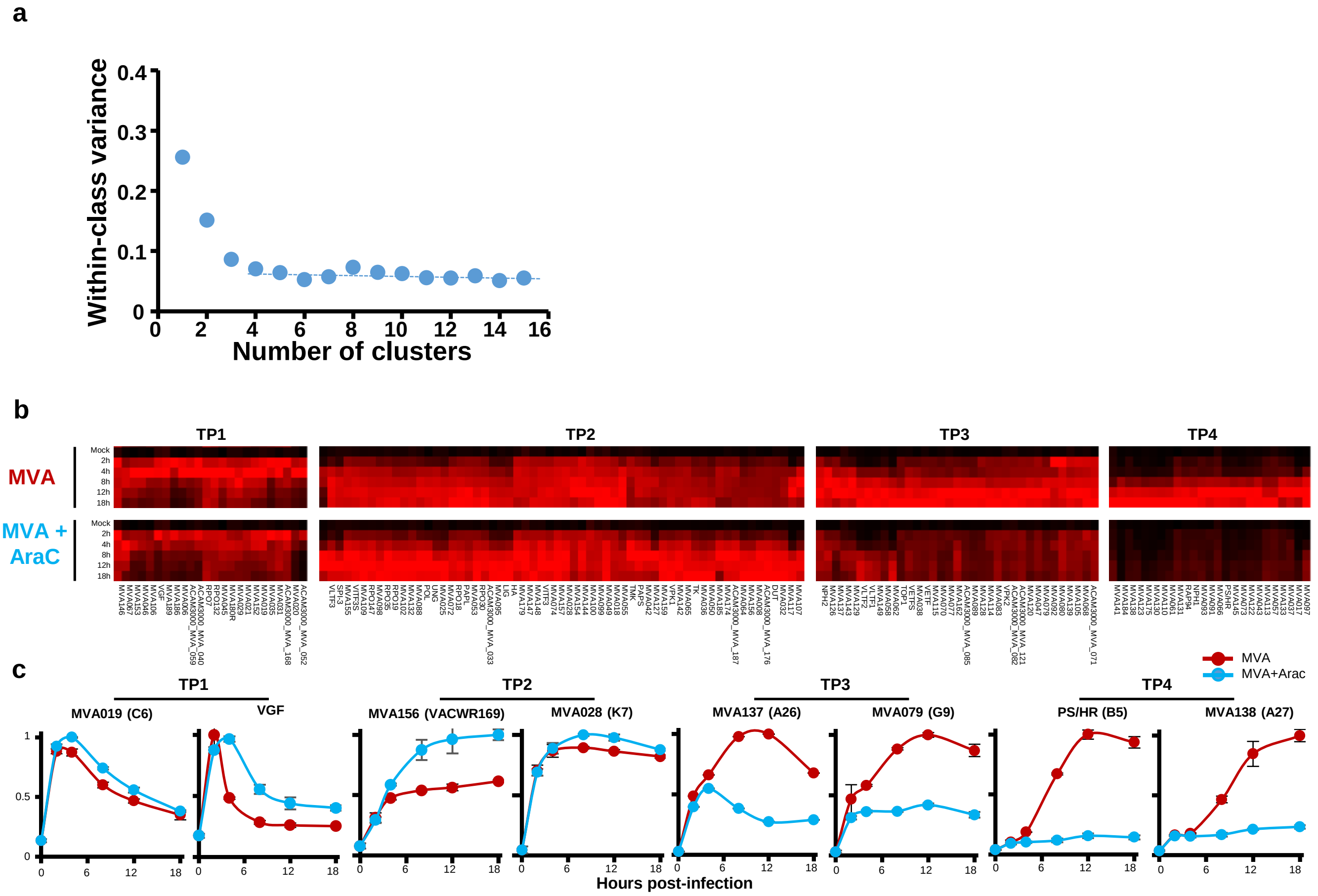

Supplementary Figure 9 | Temporal classes of MVA expression. a Number of temporal classes of MVA gene expression. The k-means method was used with 1-15 classes to cluster viral proteins quantified in HFFF-TERTs, and the summed distance of each protein from its cluster centroid was calculated. Although this summed distance necessarily becomes smaller as more clusters are added, the rate of decline decreases with each added group, eventually settling at a fairly constant rate of decline that reflects overfitting; clusters added prior to this point reflect underlying structure in the temporal protein data, whereas clusters subsequently added through overfitting are not informative. The point of inflexion fell between four and six classes, suggesting that there are at least four distinct temporal protein profiles of viral protein expression. **b** Viral protein were clustered into 4 classes defined by the k-means method. Proteins were subsequently clustered hierarchically within each class. **c** Examples of each class. Protein names in brackets indicate orthologous proteins from VACV-WR (**Supplemental Table 4**). Error bars = range.
